# Critical Rheumatic Mitral Stenosis Unmasked During Pregnancy Complicated by Preeclampsia With Severe Features and Managed With Urgent Balloon Mitral Valvuloplasty

**DOI:** 10.7759/cureus.115421

**Published:** 2026-08-29

**Authors:** Umama Gorsi, Nathan Kim, Rishi Sukhija, Julia Kumar, Pooneh Nabavizadeh

**Affiliations:** 1 Cardiology, University System of Maryland, Baltimore, USA; 2 Internal Medicine, Harbor–UCLA Medical Center, Torrance, USA; 3 Cardiology, University of Cincinnati Medical Center, Cincinnati, USA; 4 Internal Medicine, University of Cincinnati Medical Center, Cincinnati, USA

**Keywords:** mitral valve, pregnancy, pulmonary edema, rheumatic heart disease, stenosis

## Abstract

Pregnancy imposes significant hemodynamic changes that can unmask previously undiagnosed cardiovascular disease and complicate management when concurrent obstetric conditions are present. We present the case of a 32-year-old immigrant woman at 21 weeks’ gestation with a limited medical history due to a significant language barrier, who presented with hypertensive crisis and was diagnosed with preeclampsia with severe features. During her evaluation, she developed worsening dyspnea and acute respiratory decompensation. Further cardiac assessment revealed critical rheumatic mitral stenosis, accompanied by severe left atrial enlargement and markedly elevated transmitral gradients. The coexistence of preeclampsia with severe features and critical mitral stenosis posed a complex hemodynamic challenge, leading to flash pulmonary edema and progressive maternal instability.

Given the high maternal and fetal risk associated with continued deterioration, a multidisciplinary team involving maternal-fetal medicine, cardiology, interventional cardiology, cardiac anesthesia, and critical care was assembled to determine the optimal treatment strategy. The patient underwent urgent balloon mitral valvuloplasty with atrial septostomy, resulting in marked hemodynamic improvement and resolution of pulmonary edema. Following the procedure, her clinical status stabilized, and she was discharged with close cardiology follow-up. This report highlights the importance of maintaining a high index of suspicion for underlying structural heart disease in pregnant patients presenting with pulmonary edema, particularly when symptoms appear disproportionate to the severity of preeclampsia. It also demonstrates the critical role of multidisciplinary collaboration and timely percutaneous intervention in achieving favorable maternal outcomes in the setting of severe rheumatic mitral stenosis during pregnancy.

## Introduction

Rheumatic mitral stenosis remains a significant cause of cardiovascular morbidity during pregnancy and may be unmasked by the physiological hemodynamic changes associated with gestation. Patients with severe mitral stenosis are at increased risk of pulmonary edema, supraventricular arrhythmias, heart failure, and maternal mortality, particularly in the presence of additional obstetric complications [1,2]. Preeclampsia with severe features further increases cardiovascular stress and may precipitate decompensation in patients with previously undiagnosed valvular disease. Although rheumatic mitral stenosis is uncommon in developed countries, it remains an important cause of cardiovascular disease among immigrant and underserved populations [1]. We present a case of critical rheumatic mitral stenosis diagnosed during the evaluation of preeclampsia with severe features in a pregnant patient, whose clinical course was complicated by flash pulmonary edema requiring emergent balloon mitral valvuloplasty and atrial septostomy.

## Case presentation

A 32-year-old G5P2022 Micronesian immigrant woman at approximately 21 weeks' gestation presented to the local health department to establish prenatal care and was referred to the emergency department after being found to have a blood pressure of 222/132 mmHg. She reported a several-day history of headaches and severe exertional dyspnea, consistent with New York Heart Association (NYHA) class IV symptoms. Physical examination was notable for a grade III/VI diastolic murmur. Her medical history was difficult to obtain because of a significant language barrier. She spoke Pohnpeian, a language with limited interpreter availability in the United States. She reported two prior miscarriages associated with elevated blood pressure, but had never been formally diagnosed with hypertension and had no established primary care provider.

Initial management included intravenous magnesium sulfate and nifedipine for preeclampsia with severe features. Given the severity of her dyspnea, NYHA class IV symptoms, and the presence of a grade III/VI diastolic murmur on cardiac examination, a cardiac etiology was suspected in addition to preeclampsia-related cardiopulmonary complications. This prompted further cardiac evaluation, including measurement of brain natriuretic peptide (BNP) and transthoracic echocardiography (TTE). BNP was elevated at 678 pg/mL (normal: < 100 pg/mL). TTE revealed critical rheumatic mitral stenosis, with a mitral valve area of 0.6 cm² and a mean transmitral gradient of 22 mmHg. The left atrium was severely enlarged, with bowing of the atrial septum, and the estimated pulmonary artery systolic pressure was 60 mmHg (Table 1).

**Table 1:** Echocardiographic and hemodynamic findings TTE: transthoracic echocardiography; TEE: transesophageal echocardiography; LA: left atrial

Parameter	Patient value	Reference range
Mitral valve area (TTE)	0.6 cm²	4–6 cm²
Mean transmitral gradient (TTE)	22 mmHg	< 2 mmHg
Left atrial size	Severely enlarged	Normal LA volume index < 34 mL/m²
Estimated pulmonary artery systolic pressure	60 mmHg	< 35 mmHg
Right atrial pressure	12 mmHg	2–8 mmHg
Pulmonary capillary wedge pressure	38 mmHg	6–12 mmHg
Pulmonary artery pressure	100/40 mmHg	Approximately 15–30/4–12 mmHg
Mitral valve area (pre-valvuloplasty TEE)	0.3 cm²	4–6 cm²
Left atrial pressure (pre-valvuloplasty)	40 mmHg	4–12 mmHg
Mean transmitral gradient (post-valvuloplasty)	3.87 mmHg	< 2 mmHg
Left atrial pressure (post-valvuloplasty)	20 mmHg	4–12 mmHg
Wilkins score	13	≤ 8 generally favorable for balloon valvuloplasty

Given the diagnosis of preeclampsia with severe features before fetal viability, complicated by pulmonary edema and newly diagnosed critical mitral stenosis, maternal-fetal medicine recommended urgent pregnancy termination because of the substantial risk of maternal morbidity and mortality. Before the planned dilation and evacuation (D&E), the patient developed acute hypoxic respiratory failure secondary to pulmonary edema, requiring bilevel positive airway pressure and intravenous diuresis. Right heart catheterization demonstrated markedly elevated filling pressures, including a pulmonary capillary wedge pressure of 38 mmHg, pulmonary artery pressure of 100/40 mmHg, and right atrial pressure of 12 mmHg, with preserved cardiac output. Following D&E, transesophageal echocardiography (Figure 1) confirmed severe rheumatic mitral stenosis, with a valve area of 0.6 cm², a mean diastolic gradient of 19 mmHg, severe thickening and calcification of the mitral leaflets, severe pulmonary hypertension, moderate tricuspid regurgitation, and a Wilkins score of 13 (Table 1).

**Figure 1:**
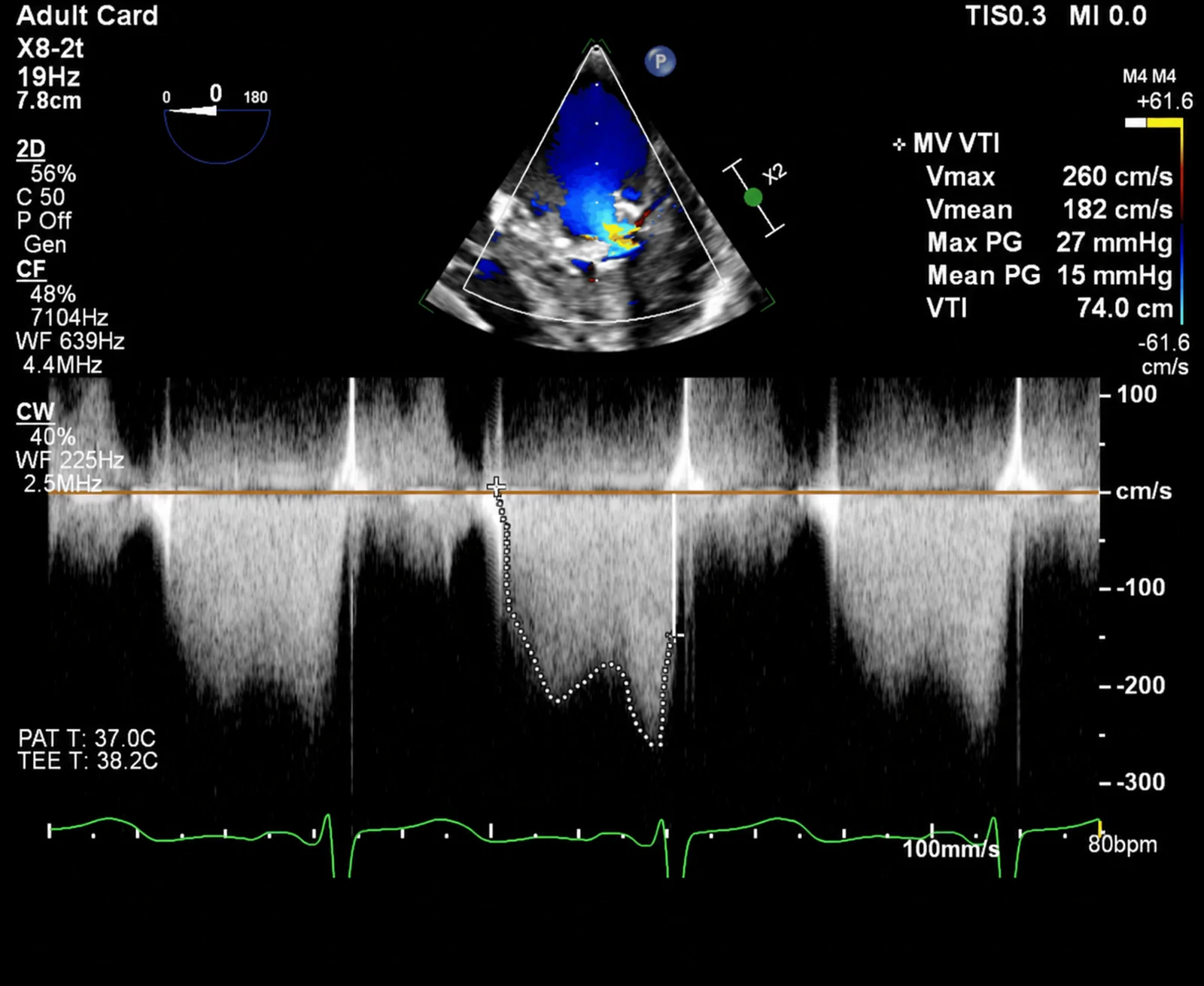
Pre-procedural TEE Doppler Pre-procedural TEE continuous-wave Doppler assessment demonstrating severe rheumatic mitral stenosis with a mean transmitral gradient of 15 mmHg prior to balloon mitral valvuloplasty TEE: transesophageal echocardiography

Despite aggressive medical therapy with beta-blockers, vasodilators, and diuretics, the patient experienced recurrent pulmonary edema and progressive respiratory failure. On hospital day eight, she developed severe hypoxemia, with oxygen saturation falling below 55%, following episodes of supraventricular tachyarrhythmia. She required intubation and mechanical ventilation. Due to hemodynamic instability, she was deemed a poor candidate for immediate surgical valve replacement. After multidisciplinary discussion involving cardiology, cardiac surgery, critical care, maternal-fetal medicine, and structural heart specialists, emergent percutaneous balloon mitral valvuloplasty was performed. Venoarterial extracorporeal membrane oxygenation was considered as rescue therapy in the event of procedural deterioration. Preprocedural transesophageal echocardiography (Figure 1) demonstrated a mitral valve area of 0.3 cm² and a left atrial pressure of 40 mmHg. Balloon mitral valvuloplasty with atrial septostomy resulted in marked hemodynamic improvement, with a reduction in the mean transmitral gradient to 3.87 mmHg and left atrial pressure to 20 mmHg (Figure 2).

**Figure 2:**
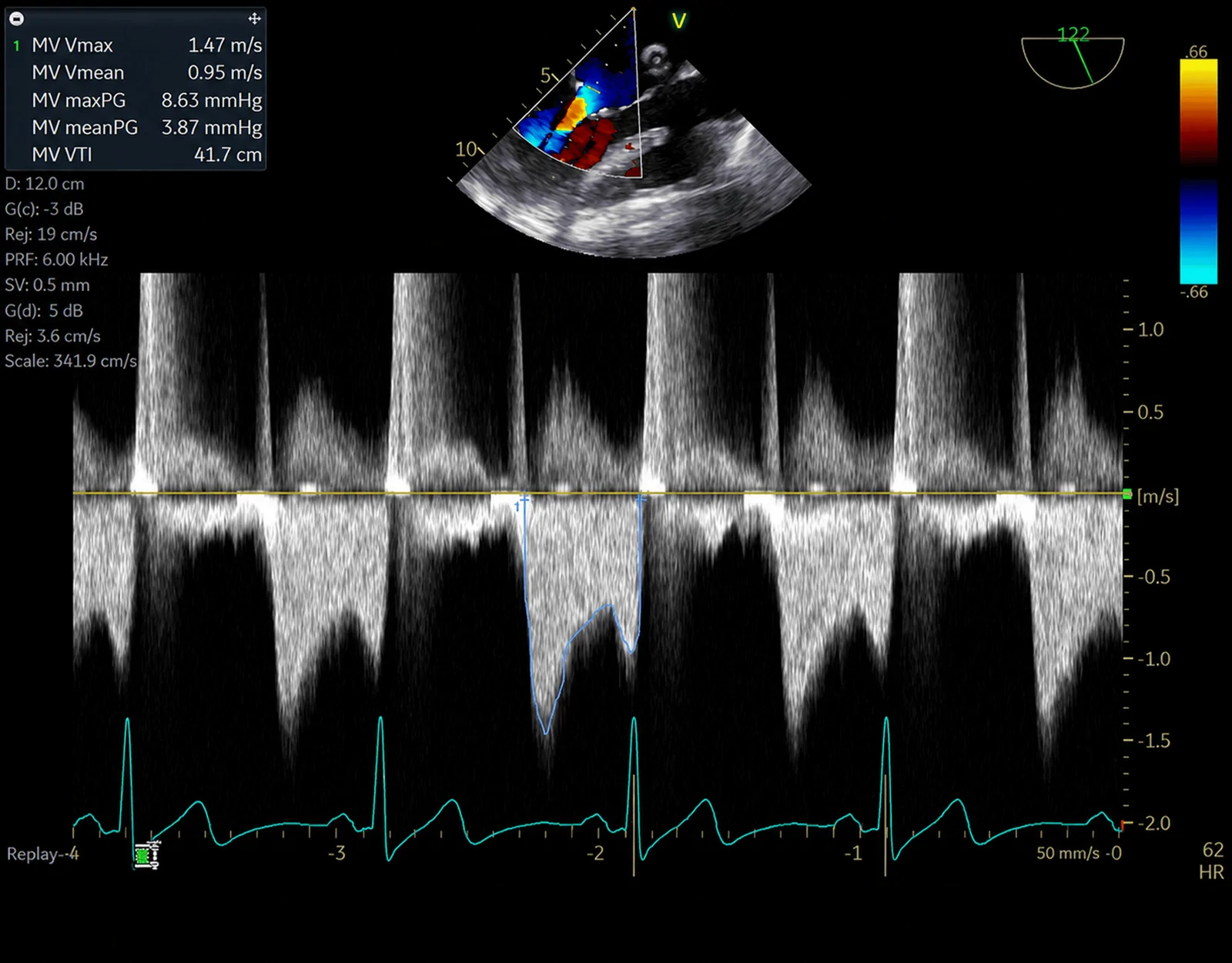
Post-procedural TEE Doppler Post-procedural TEE continuous-wave Doppler demonstrating successful balloon mitral valvuloplasty with reduction in mean transmitral gradient to 3.87 mmHg following intervention and atrial septostomy TEE: transesophageal echocardiography

The patient's respiratory and hemodynamic status improved significantly after the intervention. She was discharged on hospital day 16 on carvedilol, furosemide, valsartan, and long-acting reversible contraception. At one-month follow-up, she reported NYHA class I symptoms and remained clinically stable. However, she was subsequently lost to follow-up due to financial constraints, language barriers, and difficulties accessing interpreter services.

## Discussion

Mitral stenosis is the most common clinically significant valvular lesion encountered during pregnancy. The physiological increases in plasma volume, heart rate, and cardiac output during pregnancy can precipitate decompensation in patients with previously asymptomatic or undiagnosed disease [1,2]. Severe mitral stenosis may lead to elevated left atrial pressures, pulmonary hypertension, pulmonary edema, and atrial arrhythmias, all of which contribute to increased maternal risk [1,2].

This case illustrates the complex interaction between preeclampsia with severe features and critical rheumatic mitral stenosis. The patient's preeclampsia initially prompted medical evaluation, ultimately leading to the diagnosis of advanced rheumatic valvular disease. Pregnancy termination was recommended because of the substantial maternal risk associated with previable preeclampsia, pulmonary edema, and critical mitral stenosis. Despite termination, the hemodynamic shifts associated with the postpartum period further exacerbated her valvular disease, resulting in recurrent pulmonary edema and respiratory failure. These postpartum changes have long been recognized as a period of heightened cardiovascular risk due to rapid increases in preload and autotransfusion from the contracting uterus [3].

Current management strategies for severe symptomatic mitral stenosis emphasize heart rate control, cautious diuresis, and intervention when symptoms persist despite optimal medical therapy [4]. Although this patient had unfavorable valve anatomy, with a Wilkins score of 13, emergent balloon mitral valvuloplasty was pursued because of life-threatening clinical deterioration and prohibitive surgical risk. Current ESC guidelines recommend percutaneous balloon mitral valvuloplasty in symptomatic patients with severe mitral stenosis who remain unstable despite optimal medical therapy [4]. The procedure resulted in substantial reductions in the transmitral gradient and left atrial pressure, accompanied by rapid clinical improvement. Prior studies have demonstrated high procedural success rates and favorable maternal outcomes following percutaneous balloon mitral valvuloplasty during pregnancy [5].

An additional important aspect of this case is the impact of social determinants of health. The patient's immigrant status, limited access to healthcare, lack of prenatal care, financial barriers, and language difficulties contributed to delayed diagnosis and presented challenges throughout hospitalization and follow-up. Rheumatic heart disease continues to disproportionately affect vulnerable and underserved populations, even within developed healthcare systems [6]. Recognition of these barriers is critical for improving outcomes and ensuring equitable cardiovascular care. Migration, limited healthcare access, language barriers, and socioeconomic disparities have all been identified as factors contributing to delayed diagnosis and management of rheumatic heart disease [7].

## Conclusions

Critical rheumatic mitral stenosis may remain undiagnosed until the hemodynamic stress of pregnancy precipitates severe clinical decompensation. In this case, preeclampsia with severe features led to the discovery of advanced rheumatic mitral stenosis, while postpartum physiological changes contributed to recurrent pulmonary edema and respiratory failure. Emergent balloon mitral valvuloplasty and atrial septostomy resulted in successful hemodynamic stabilization despite unfavorable valve anatomy. This report underscores the importance of multidisciplinary management, early recognition of structural heart disease in pregnant patients presenting with cardiopulmonary symptoms, and consideration of social determinants of health that may contribute to delays in diagnosis and treatment.
